# Bilateral impending macular holes after a high-voltage electrical shock injury and its surgical outcome: a case report

**DOI:** 10.1186/1752-1947-8-399

**Published:** 2014-12-02

**Authors:** Pingbo Ouyang, Anushavan Karapetyan, Juanlian Cui, Xuanchu Duan

**Affiliations:** Department of Ophthalmology, The Second Xiangya Hospital, Central South University, 139 Renmin Middle Road, Changsha, Hunan 410011 China

**Keywords:** Electrical shock injury, Macular hole, Optical coherence tomography

## Abstract

**Introduction:**

A macular hole is a rare complication after high-voltage electrical shock injury and only a few cases have been reported to date. To the best of our knowledge, this is the first report of bilateral impending macular holes after high-voltage electrical shock.

**Case presentation:**

We report a case of bilateral impending macular holes in a 39-year-old male Chinese patient who sustained a high-voltage electrical shock three months prior to presentation. Our patient complained of gradually diminished eyesight in both eyes, with visual acuity of 20/100 and 20/40 in his right and left eyes respectively. Our patient underwent pars plana vitrectomy accompanied by octafluoropropane gas and triamcinolone acetonide injections, and was discharged from our hospital with slightly improved vision.

**Conclusion:**

The visual outcome of impending macular holes caused by high-voltage electrical shock may be poor despite tissue residue at the fovea and surgical intervention aimed at aiding macular recovery. Surgery is, however, effective in the short term in restoring normal anatomical macular structure.

## Introduction

Ocular complications after electrical shock injuries were first reported in 1722 by St. Yves, who described cataract development in a patient struck by lightning [1]. High-voltage injury is a special type of widely occurring trauma that usually leads to serious physical damage. The severity is closely related to the voltage power, electrical current intensity, polarization and contact duration [2]. High-voltage wounding may lead to various ocular pathologies, including eyelid skin burns, iridocyclitis, electric cataracts, macular edema, optic neuropathy and, rarely, macular holes [3]. To the best of our knowledge, there are only three reports of a macular hole following high-voltage electrical injuries [4–6] and this is the first report of impending macular holes after electrical shock. We report a case of bilateral impending macular holes caused by a high-voltage electrical injury and intend to highlight the effectiveness of the surgical treatment in a short-term postoperative period.

## Case presentation

A 39-year-old male Chinese patient presented to our hospital complaining of progressively decreased vision in his right eye, relating it to a high-voltage electrical injury occurring three months before. The accident occurred upon completion of his work under 35KV high-voltage wires. In his words, after straightening up from a prostrate posture, he immediately felt his body being pulled upward to the wires and was struck instantly. Because of the subsequent muscular contraction induced by the strike, he hit his right hip on a hard object nearby, after which his body was intercepted and he fell from the platform. He was immediately taken to the local hospital by ambulance in an unconscious state and admitted to an intensive care unit (ICU) with a diagnosis of multiple systemic skin burns and blunt traumas caused by electrical shock. Two days later, he regained consciousness and, apart from other systemic signs, tearing watery red eyes were noted by the ICU doctors. This condition was not considered to be severe and, without an ophthalmological consultation, our patient was discharged at a later date.

During the period of hospitalization and recuperation at home, our patient did not pay attention to a slight distortion in his eyesight. However, after returning to work, and two weeks before presenting to our hospital, he found his vision gradually worsening.

**Figure 1:**
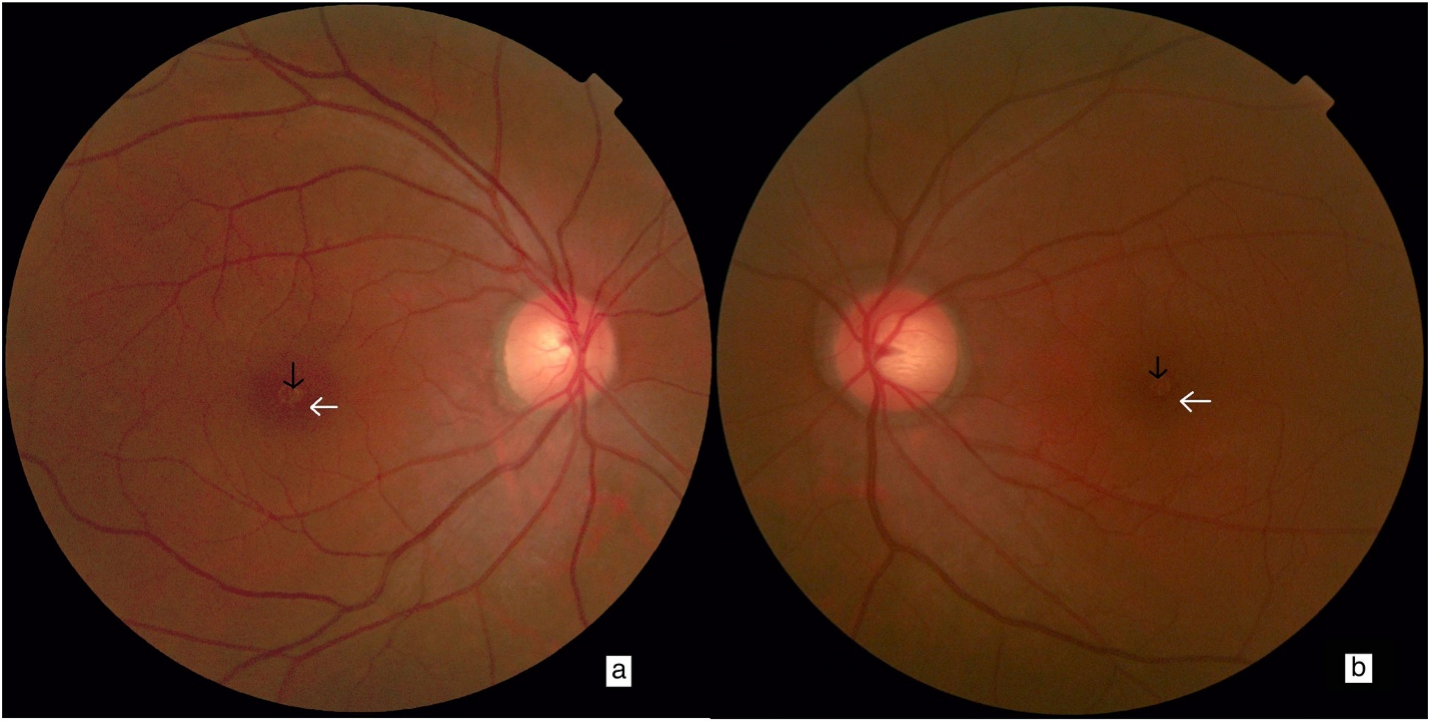
**Preoperative fundus color photos.** In **(a)** right and **(b)** left eyes, macular holes (black arrows) and circular lesions, so-called cuffs (white arrows), are visible.

**Figure 2:**
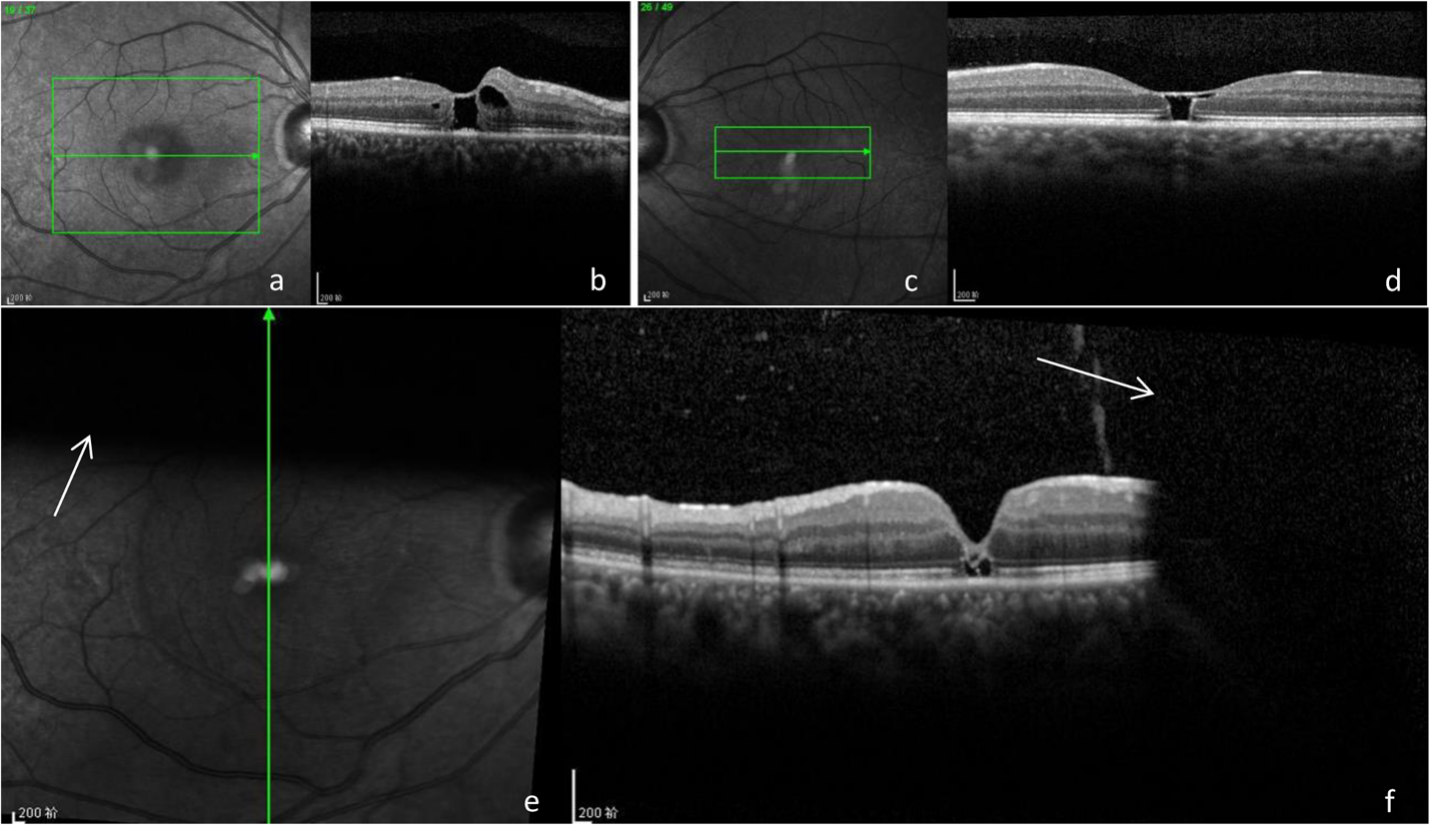
**Pre- and postoperative images.** Red free fundus images of the **(a)** right and **(c)** left eyes. Spectral domain optical coherence tomography (SD-OCT) images of **(b)** right and **(d)** left eyes. The green box corresponds to the area examined and the green arrow represents the retinal cross section. **(e)** Postoperative red free fundus image with **(f)** respective SD-OCT image. The green arrows represent the corresponding section scanned, and the white arrows indicate the shadowing effect from the gas.

At our hospital, a physical examination revealed multiple skin burns and scarring, especially around his right hip. His visual acuity was 20/100 in his right eye and 20/40 in his left. His intraocular pressure was measured using a non-contact puff tonometer, revealing a pressure of 13mmHg in his right eye and 15mmHg in his left. Slit lamp examination found the following in both eyes: transparent cornea, hyperemic conjunctiva, no keratic precipitates, normal anterior chamber depth, negative Tyndall sign, normal-shaped iris, no adhesions around the pupil, anterior subcapsular opacities in the lens, and vitreous detachment. Indirect ophthalmoscopy showed normal optic discs with clear boundaries, retinal hemorrhages and a well-defined ‘cuff’ at the macula (Figure 1). Spectral domain optical coherence tomography (SD-OCT) examination revealed a disruption of the retinal layers at the fovea, with a thin remainder of the internal limiting membrane, the so-called roof, surrounded by obviously edematous macula (Figure 2b,d). A diagnosis of impending macular hole and electric cataract was established.

Our patient underwent pars plana vitrectomy in his right eye, with internal limiting membrane peeling, C3F8 gas tamponade with face-down positioning and, finally, an intraocular injection of 0.05ml (2mg) triamcinolone acetonide (TA). One week postoperatively, the macular hole was closed with one third gas residue in his vitreous cavity (Figure 2e,f); his visual acuity was 20/63. We obtained our patient’s consent prior to the procedure.

## Discussion

The detailed pathophysiology of electrical injury is not yet well understood. Most injuries caused by contact with high-voltage power lines are thought to be thermal and few histologic studies have revealed coagulation necrosis consistent with thermal injury [7].

The nature and severity of electrical burn injury are directly proportional to the current strength, resistance, and duration of current flow [2]. There are two types of circuit, direct current (DC) and alternating current (AC), which affect the nature and severity of electrical injury. High-voltage DC contact causes a single muscular spasm, usually throwing the victim from the source. AC exposure to the same voltage is three times more dangerous than DC. The most destructive indirect injury occurs when a victim becomes part of an electric arc, which is a current spark formed between two objects of different potentials that are not in direct contact with each other, usually a highly charged source and a ground [8]. The very high temperature of an electric arc (around 2500°C) causes very deep thermal burns at the contact points on the skin [9]. In the case we present, a high-voltage arc was created between our patient and the power lines, leading to electrical shock. After this, an electric discharge occurred when our patient’s right hip hit the object and grounded his body, forming a high-voltage current loop between the wire, air, his body and the earth, and our patient was thrown away by the blast. The severe skin burns on our patient’s right hip indicate the exit or ground contact point, supporting the theory of the loop.

SD-OCT played a very important role in establishing the diagnosis of the impending macular hole. In the preoperative SD-OCT image of his right eye (Figure 2b), a foveal pseudocyst is evident, covered by a thin residue of internal limiting membrane with abnormal foveal contour and a separation of the posterior vitreous from the fovea. This contributed to establishing a diagnosis of cystoid macular edema and impending macular hole. In the SD-OCT image of his left eye (Figure 2d), a foveal pseudocyst with hyper-reflective and rigid walls can also be seen, which was probably caused by glial tissue formed at the edges of the hole. This was one of the reasons that surgery was only performed on his right eye.

Retinal tears caused by high-voltage electrical shock are usually located within the macular area, which has been hypothesized to be related to several factors. First, macular holes may be a result of a localized elevation in temperature of the underlying retinal pigment epithelium (RPE), causing thermal damage to the overlying retina [10]. Second, the RPE is thicker and more tightly packed in the submacular than in any other region of the eye, thus accumulating more thermal energy and heat, leading to thermal damage of the macula [4].

At completion of the surgery, 2mg of TA was injected to suppress inflammatory reactions. One week later, our patient’s visual acuity slightly increased to 20/63. SD-OCT examination showed a decrease in retinal edema, a significantly smaller macular hole and a minor cyst under the fovea.

## Conclusion

The visual outcome of impending macular holes caused by high-voltage electrical shock may be poor despite tissue residue at the fovea and surgical intervention aimed at aiding macular recovery. SD-OCT plays an important role in diagnosing an impending macular hole after electrical shock injury. In the short term, surgery proves to be effective in restoring normal macular structure.

## Consent

Written informed consent was obtained from the patient for publication of this case report and accompanying images. A copy of the written consent is available for review by the Editor-in-Chief of this journal.

## Abbreviations

AC: alternating circuit
DC: direct circuit
ICU: intensive care unit
RPE: retinal pigment epithelium
SD-OCT: spectral domain optical coherence tomography
TA: triamcinolone acetonide.
